# Mesenchymal Stem Cells Attract Endothelial Progenitor Cells via a Positive Feedback Loop between CXCR2 and CXCR4

**DOI:** 10.1155/2019/4197164

**Published:** 2019-12-05

**Authors:** Yingyun Tan, Linjing Shu, Peng Xu, Shi Bai

**Affiliations:** ^1^Stomatological Hospital of Chongqing Medical University, Chongqing, China; ^2^Chongqing Key Laboratory of Oral Diseases and Biomedical Sciences, Chongqing, China; ^3^Chongqing Municipal Key Laboratory of Oral Biomedical Engineering of Higher Education, Chongqing, China

## Abstract

Mesenchymal stem cells (MSCs) can attract host endothelial progenitor cells (EPCs) to promote vascularization in tissue-engineered constructs (TECs). Nevertheless, the underlying mechanism remains vague. This study is aimed at investigating the roles of CXCR2 and CXCR4 in the EPC migration towards MSCs. *In vitro*, Transwell assays were performed to evaluate the migration of EPCs towards MSCs. Antagonists and shRNAs targeting CXCR2, CXCR4, and JAK/STAT3 were applied for the signaling blockade. Western blot and RT-PCR were conducted to analyze the molecular events in EPCs. *In vivo*, TECs were constructed and subcutaneously implanted into GFP^+^ transgenic mice. Signaling inhibitors were injected in an orientated manner into TECs. Recruitment of host CD34^+^ cells was evaluated by immunofluorescence. Eventually, we demonstrated that CXCR2 and CXCR4 were both highly expressed in migrated EPCs and indispensable for MSC-induced EPC migration. CXCR2 and CXCR4 strongly correlated with each other in the way that the expression of CXCR2 and CXCR2-mediated migration depends on the activity of CXCR4 and vice versa. Further studies documented that both of CXCR2 and CXCR4 activated STAT3 signaling, which in turn regulated the expression of CXCR2 and CXCR4, as well as cell migration. In summary, we firstly introduced a reciprocal crosstalk between CXCR2 and CXCR4 in the context of EPC migration. This feedback loop plays critical roles in the migration of EPCs towards MSCs.

## 1. Introduction

Mesenchymal stem cell- (MSC-) based tissue-engineered constructs (TECs) have exhibited advantages for treating large bone defects [1]. However, one of the key hurdles constraining their application is insufficient blood supply after implantation [2]. Local vascular disruption imposes limits on nutrient transport and waste removal, resulting in the death of implanted MSCs [3]. Even so, bone and vessel reconstruction is significantly facilitated within TECs rather than blank scaffolds [1, 4]. Angiogenesis is closely coupled with osteogenesis and serves as a prerequisite for bone regeneration, underlining the importance of exogenous MSCs in revascularization [5]. Recently, many studies have concentrated on outlining the way by which implanted MSCs recruit host cells and provoke the host innate regenerative potential, including revascularization [6]. As reported, MSCs can promote the migration of endothelial progenitor cells (EPCs) and vessel formation via paracrine actions, which greatly expands knowledge on the repairing mechanism of MSC-based tissue engineering strategies [4]. However, the relative mechanism remains unclear.

Chemokines and their receptors play critical roles in cell migration. Therein, CXC chemokines are widely expressed by MSCs, and the inflammatory microenvironment forces the release of CXCR2 ligands in large quantities, including CXCL1/2/3, CXCL7, and CXCL8 [7]. CXCR2 plays important roles in the mobilization of EPCs. In CXCR2 knockout mice, the amounts of circulating EPCs are reduced in the bone marrow and peripheral blood, accompanied by delayed vasculogenesis [8]. Similar to CXCL8, CXCL12 is spontaneously produced by MSCs. The binding of CXCL12 to its cognate receptor CXCR4 is also crucial to the mobilization of EPCs. Local administration of CXCL12 in a distraction osteogenesis mouse model enhances the homing of EPCs to induce blood flow [9]. Recent findings confirm that the process of EPC homing, including mobilization, recruitment, and adhesion, can be regulated by CXCL1, CXCL12, and their respective receptors, CXCR2 and CXCR4 [10, 11]. All of these clues lead to a possibility that there may be a close relationship between CXCR2 and CXCR4 regarding movement of EPCs.

Thus, we set out to explore the roles and interaction of CXCR2 and CXCR4 in regulating the MSC-induced EPC migration in greater detail. We described the novel finding that the migration of EPCs towards MSCs required simultaneous activation of CXCR2 and CXCR4. A new reciprocal crosstalk between CXCR2 and CXCR4 was discovered. This crosstalk linked, acted via signal transducers and activators of transcription-3 (STAT3), and accounted for the MSC-induced EPC migration.

## 2. Materials and Methods

### 2.1. Cell Isolation and Culture

All protocols involving human subjects were approved by the Stomatological Hospital of Chongqing Medical University, with all subjects providing informed consent. Human bone marrow MSCs (hBMSCs) were isolated and cultured as previously described [7]. They were cultured in basic culture medium (BCM) containing Dulbecco's modified Eagle's medium/F12 (DMEM/F12; 1 : 1; Hyclone, USA), 10% heat-inactivated fetal bovine serum (FBS; Gibco, USA), and 100 U/ml penicillin/streptomycin (Gibco, USA). Cells were grown at 37°C in a humidified atmosphere of 5% CO_2_ and routinely passaged while reaching 80-90% confluency. hBMSCs at passage 4 were harvested for experiments. Phenotypic characterization by FACS showed that cells were homogenously positive for the cell surface antigens CD44, CD73, CD90, and CD105 and negative for CD14, CD34, and CD45 (Supplementary [Supplementary-material supplementary-material-1]).

Human endothelial progenitor cells (hEPCs) were purchased from the American Type Culture Collection (ATCC number PCS-800-012). Cells were cultured in an Endothelial Cell Medium (ECM; ScienCell Research Laboratories, Canada) containing 10% FBS and 100 U/ml penicillin/streptomycin at 37°C in 95% humidified air. The medium was changed every other day. Cells were routinely passaged while reaching 80-90% confluency. Cells were homogeneously CD34^+^, CD133^+^, and VEGFR2^+^ (Supplementary [Supplementary-material supplementary-material-1]).

All animal manipulations were approved by the Institutional Animal Care and Use Committee of the Chongqing Medical University. Mouse bone marrow MSCs (mBMSCs) were isolated and cultured as described previously [12]. Briefly, bone marrow cells were harvested by flushing nucleated cells out of murine femora using phosphate-buffered saline (PBS). After centrifugation, cells were cultured in DMEM/F12 supplemented with 15% FBS and 100 U/ml penicillin/streptomycin for 24 hours. Subsequently, nonadherent cells were removed, and the medium was replaced by fresh medium and changed every 2-3 days. Retained cells were defined as passage zero (P0) cells and routinely passaged while reaching 80-90% confluency. Cells at passage 4 were harvested for further use.

### 2.2. Gene Transfection

For gene knockdown, shRNA lentiviral particles targeting CXCR2, CXCR4, and STAT3 were purchased from Santa Cruz Biotechnology (USA). For gene transfection, EPCs (1 × 10^5^) were seeded in six-well plates and grown overnight at the logarithmic growth phase. Then, they were transfected with shRNA lentivirus particles or negative controls by adding particles into a culture medium containing 5 *μ*g/ml Polybrene (Santa Cruz Biotechnology, USA) and incubated overnight at 37°C, 5% CO_2_. Stable clones expressing the virus were selected by resistance to puromycin (0.5 *μ*g/ml; Sigma-Aldrich, USA) and cultured for 48 hours before use. Reduced CXCR2, CXCR4, or STAT3 expression in selected cells was confirmed by western blot.

### 2.3. Migration Assays In Vitro

Following the method reported previously [12], hBMSCs at passage 4 were obtained and treated with BCM supplemented with 4 ng/ml interleukin- (IL-) 1*β*, 10 ng/ml IL-6, and 20 ng/ml tumor necrosis factor *α* (TNF-*α*; all from PeproTech, USA) to prepare the conditioned medium of MSCs (MSC-CM). After 48 hours, the supernatants were collected, centrifuged, aliquoted, and stored at -80°C.

Migration assays were performed in Transwell chambers with 8 *μ*m filters (Corning Costar Corp., USA). 700 *μ*l of migration-inducing medium was added into the bottom chamber. hEPCs were pretreated by different measures, as detailed in Table 1. Then, hEPCs were loaded into the upper chambers (1 × 10^4^ cells/chamber). After 8-hour migration at 37°C, hEPCs were collected for protein and RNA analysis. Meanwhile, nonmigrating cells on the upper side of the filter were removed with cotton wool swabs (Norgen Biotek, Canada). Migrated cells on the lower side were washed with PBS 3 times and fixed with 4% paraformaldehyde (Boster Biological Technology, Wuhan, China). Then, they were stained with 4′,6-diamidino-2-phenylindole (DAPI; Invitrogen, USA) and counted using a microscope. For each group, the number of migrated cells was counted on 10 random high-power fields (200x magnification) and averaged. Migration assay was separately conducted 3 times.

### 2.4. Western Blot Assay

Total cellular protein was extracted using a lysis buffer (100 mM Tris at pH 8.0, 10% glycerol, and 1% SDS). After protein concentration was determined by a NanoVue spectrophotometer (GE, USA), an equal amount of protein samples (30 *μ*g) was loaded on sodium dodecyl sulfate-polyacrylamide gel electrophoresis (SDS-PAGE, Beyotime, China) and subsequently transferred onto polyvinylidene difluoride membranes (Millipore, USA). The preparations were then blocked with 5% skimmed milk for 1 hour at room temperature and incubated with the following primary antibodies: anti-CXCR2, anti-CXCR4 (1 : 1000 dilution; Santa Cruz, USA), and anti-pSTAT3 (1 : 2000 dilution; Abcam, USA) overnight at 4°C. The preparations were then incubated with a horseradish peroxidase-conjugated secondary antibody (1 : 2000 dilution; Southern Biotech, Birmingham, AL) for 30 min. Signals were detected using an enhanced chemiluminescence kit (Millipore, USA) according to the manufacturer's instruction. GAPDH served as the internal control.

### 2.5. Quantitative Real-Time PCR

Total RNA was extracted using a TRIzol Reagent (Invitrogen, CA) and reverse transcribed into cDNA using a reverse transcriptase according to the user manual (Promega, USA). All reactions were performed with SYBR Green Mix (Takara, Japan). All experiments were performed in triplicate, and results were normalized to the housekeeping gene GAPDH. The primers used are shown in Table 2.

### 2.6. Migration Assays In Vivo

Based on their excellent cytocompatibility, demineralized bone matrixes (DBM; purchased from Datsing Bio-Tech Co., Ltd., Beijing, China) were used as scaffolds. TECs were fabricated according to the method previously described [13]. For each scaffold, 20 *μ*l of a single mBMSC suspension (1 × 10^6^ cells/ml) was dropwise instilled onto two opposite surfaces. After 2 hours, a culture medium was added to immerse the scaffold. The medium was changed every 3 days. TECs cultured for 7 days were collected and subcutaneously implanted into GFP^+^ transgenic mice. Signaling inhibitors, including SB225002, AMD3100, ruxolitinib, or saline (control), were injected in an orientated manner into TECs every 2 days. Groups of animals were sacrificed after 10 days. Implants were collected and subjected to RT-PCR analysis. The rest of the implants were fixed with 4% paraformaldehyde and frozen sections (8 *μ*m thick) were prepared. Sections were permeabilized with 0.3% Triton X-100 and blocked with a normal donkey serum (1 : 20; Huayueyang Biotechnology, Beijing, China). Subsequently, samples were incubated with polyclonal rabbit anti-mouse-CD34 (1 : 500; Abcam, UK) overnight at 4°C, followed by staining with donkey anti-rabbit-Cy3 (1 : 100; Jackson ImmunoResearch, USA) for 1 hour and DAPI for 10 min. Relative cellularity was evaluated with a confocal laser scan microscope (CLSM; Leica Biosystems, Germany).

### 2.7. Statistical Analysis

Results are presented as mean ± SEM. For the RT-PCR and western blot, statistical differences were analyzed by a paired Student *t*-test. For Transwell migration assays, a one-way ANOVA followed by the SNK test was conducted to determine the statistical significance between groups (SPSS version 13.0). A *p* value < 0.05 was considered statistically significant.

## 3. Results

### 3.1. Migration of EPCs Depends on the Expression and Activation of CXCR2 and CXCR4

**Figure 1:**
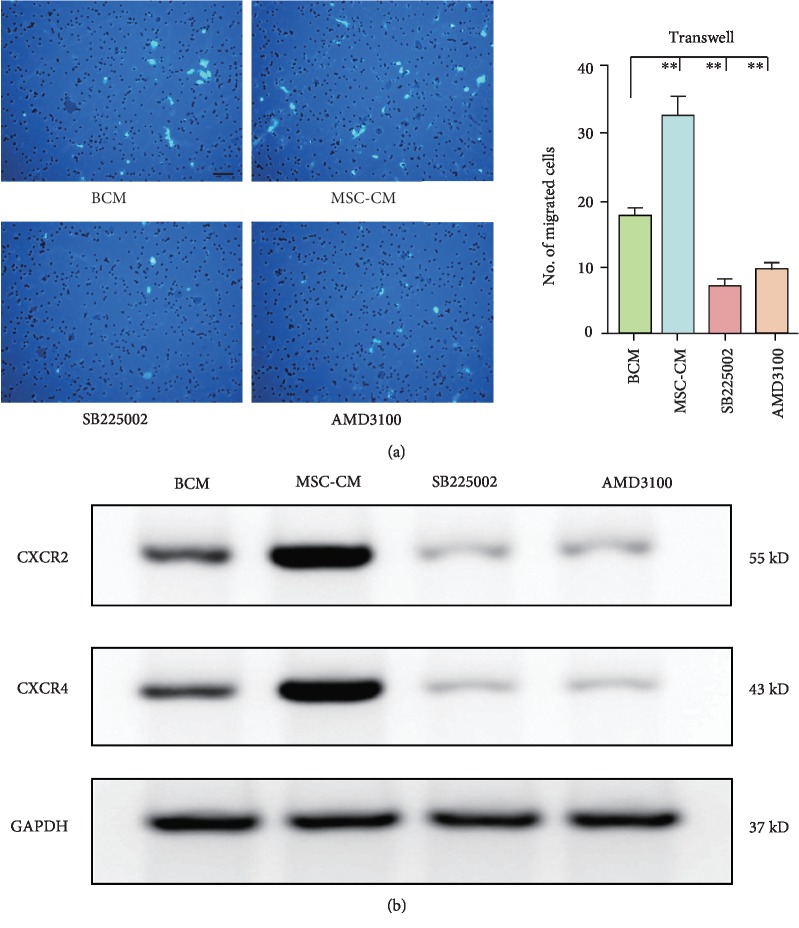
CXCR2 and CXCR4 levels were higher in the migrated EPCs induced by MSCs. (a) Representative images of migrated EPCs. The migration capacity of EPCs was observed using a Transwell culture system. Migrated EPCs were stained with DAPI. Scale bar, 50 *μ*m. The amount of migrated EPCs was quantified and presented as a bar graph (*n* = 3; ^∗∗^*p* < 0.01). (b) Changes of protein expressions of CXCR2 and CXCR4 after blocking CXCR2 or CXCR4. After migration, EPCs were collected and subjected to western blot. BCM: basic culture medium; MSC-CM: conditioned media of mesenchymal stem cells.

Attracting EPCs with MSC-CM for 8 hours facilitated cell migration (Figure 1(a)). To study the impact of CXCR2 and CXCR4 on the migration of EPCs towards MSCs, we blocked CXCR2 and CXCR4 with SB225002 and AMD3100, respectively. As revealed, EPC migration was almost abolished (Figure 1(a)). Moreover, western blot showed that CXCR2 and CXCR4 were both constitutively expressed by EPCs and upregulated in response to MSCs (Figure 1(b)). Notably, blockade of either CXCR2 or CXCR4 led to striking decreases in the expression levels of both CXCR2 and CXCR4.

### 3.2. CXCR2 and CXCR4 Cross-Activate Each Other

**Figure 2:**
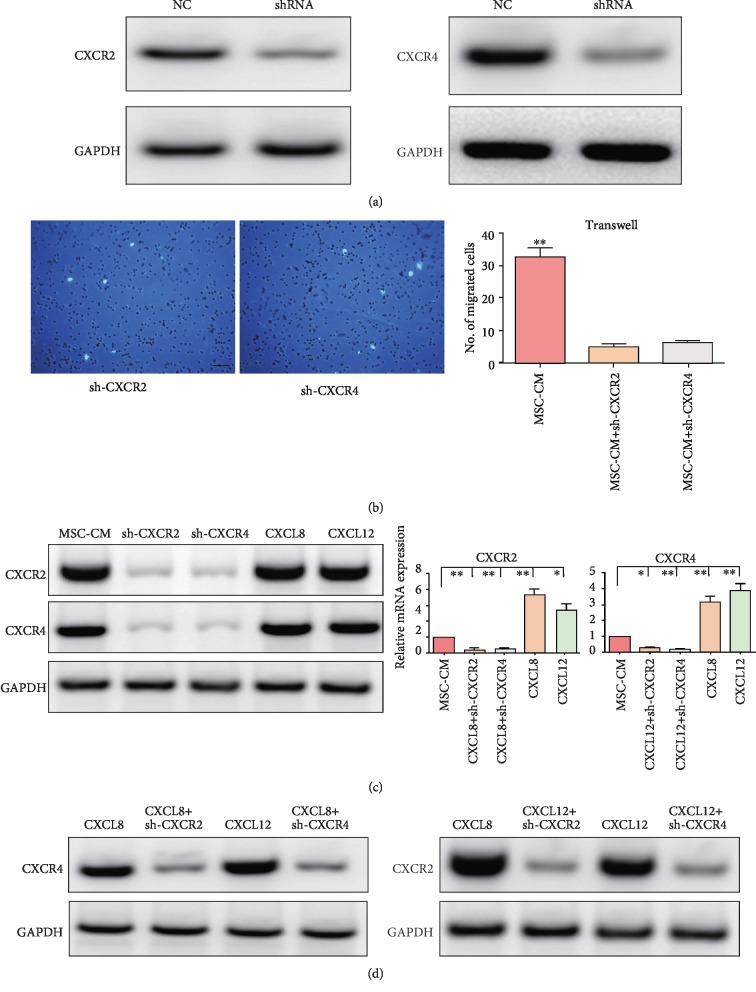
CXCR2 and CXCR4 cross-activated each other. (a) The effectiveness of shRNAs. Gene knockdown was verified by western blot. (b) Representative images of migrated EPCs. (c) Changes of protein and mRNA expressions after different treatments. ∗ in red: chemokines vs. MSC-CM; ∗ in blue: shRNA vs. MSC-CM. (d) Changes of protein expressions after different treatments. NC: negative control; MSC-CM: conditioned media of mesenchymal stem cells. Scale bar, 50 *μ*m. *n* = 3. ^∗^*p* < 0.05 and ^∗∗^*p* < 0.01.

To further investigate the relationship of CXCR2 and CXCR4 in the migration of EPCs, CXCR2 and CXCR4 were knocked down in EPCs by their respective shRNAs (Figure 2(a)). Consistent with results from antagonists, knockdown of CXCR2 exerted inhibitory effects on cell migration (Figure 2(b)) and led to a remarkable decrease in CXCR4 expression and vice versa (Figure 2(c)). Inspired by these findings, we used CXCR2 and CXCR4 ligands to stimulate EPCs. When treating cells with CXCR2 ligand CXCL8, the expressions of CXCR2 and CXCR4 were elevated simultaneously at both the protein and mRNA levels (Figure 2(c)). Moreover, the incentive compacts of CXCL8 on CXCR2 and CXCR4 expressions were impaired by not only CXCR2 knockdown but also CXCR4 (Figures 2(c) and 2(d)). Semblable variation was also observed for CXCL12 (Figures 2(c) and 2(d)).

### 3.3. A Positive Feedback Loop of CXCR2-STAT3-CXCR4 Exists in Migrated EPCs

**Figure 3:**
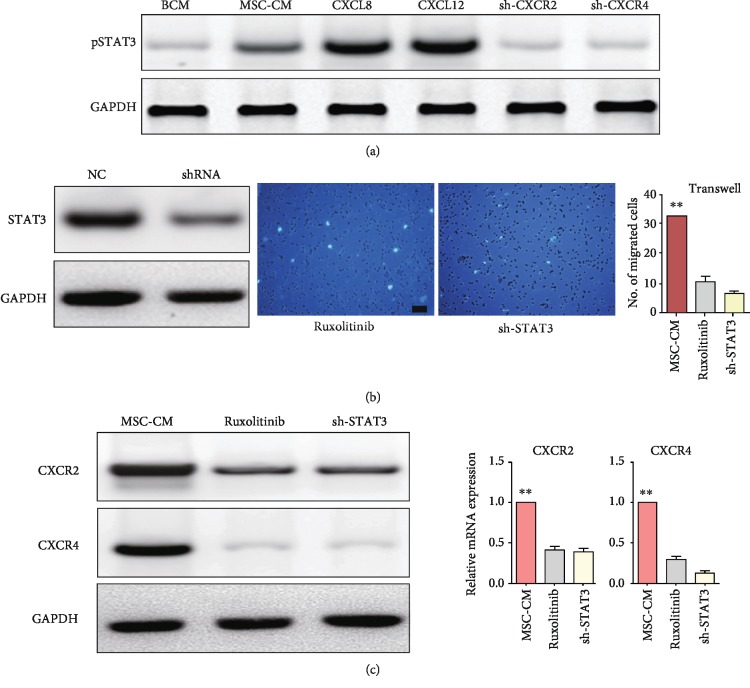
A positive feedback loop of CXCR2/CXCR4-STAT3 existed in EPCs. (a) Changes of STAT3 phosphorylation in EPCs after different treatments. (b) The effectiveness of shRNA for STAT3. Representative images of migrated EPCs. (c) Changes of protein expressions in EPCs after blockade of STAT3. BCM: basic culture medium; MSC-CM: conditioned media of mesenchymal stem cells. Scale bar, 50 *μ*m. *n* = 3. ^∗^*p* < 0.05 and ^∗∗^*p* < 0.01.

To decode how CXCR2 and CXCR4 cross-activate each other, current literature was reviewed and the JAK/STAT pathway was chosen for verification. Indeed, treating cells with MSC-CM, CXCL8, or CXCL12 significantly enhanced the phosphorylation of STAT3. Meanwhile, a substantial decrease in STAT3 phosphorylation was detected after CXCR2 or CXCR4 knockdown (Figure 3(a)). To dissect the possible positive feedback loop of the CXCR2/4-STAT3 circuit, we then explored whether STAT3 regulated CXCR2 or CXCR4 signaling in the migration of EPCs. STAT3 was knocked down in EPCs by shRNA (Figure 3(b)). Treating EPCs with the JAK/STAT3 pathway inhibitor ruxolitinib strongly suppressed MSC-CM-induced EPC migration, as well as CXCR2 and CXCR4 expressions (Figures 3(b) and 3(c)). After the STAT3 knockdown, cell migration and expressions of CXCR2 and CXCR4 were also dramatically reduced (Figures 3(b) and 3(c)).

### 3.4. The Crosstalk between CXCR2 and CXCR4 Contributes to EPC Migration

**Figure 4:**
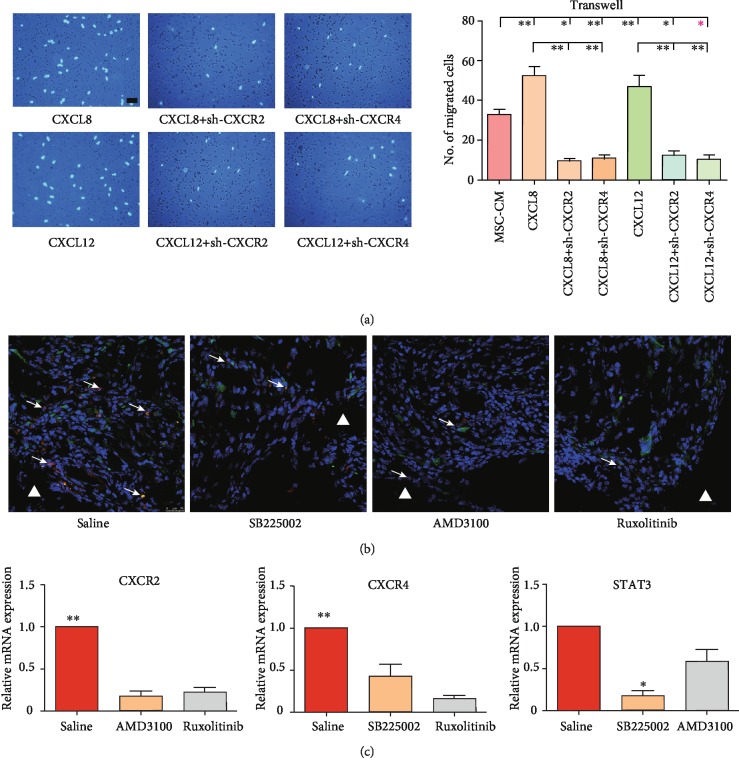
The crosstalk between CXCR2 and CXCR4 contributed to EPC migration. (a) Representative images of migrated EPCs receiving different treatments. Scale bar, 50 *μ*m. (b) Representative images of the recruited host CD34^+^ cells *in vivo* (white arrows). White triangle: implant area; white arrows: GFP^+^/CD34^+^ cells. Scale bar, 25 *μ*m. (c) Relative mRNA expressions. The quantification comparison was exhibited as bar graphs (*n* = 3; ^∗^*p* < 0.05 and ^∗∗^*p* < 0.01).

Next, we investigated how this signaling loop affected EPC migration. Treating EPCs with CXCL8 and CXCL12 significantly promoted cell migration towards MSCs (Figure 4(a)). When CXCR2 was knocked down, the promigratory effect of CXCL8 was aborted (Figure 4(a)). Intriguingly, knockdown of CXCR4 also hampered the effect of CXCL8 (Figure 4(a)), although CXCR4 was not a known receptor of CXCL8. Similarly, CXCL12-induced migration was blocked by CXCR2 knockdown (Figure 4(a)), while CXCR2 is not a receptor of CXCL12 either. Consistent with Transwell assays *in vitro*, the *in vivo* recruitment of host CD34^+^ cells (majorly endothelial-lineage progenitors) towards implanted MSCs was almost abolished by local delivery of SB225002, AMD3100, or ruxolitinib (Figure 4(b)). Moreover, CXCR2 expression levels in TECs were significantly reduced by the blockade of CXCR4 or STAT3. Similarly, the CXCR2 or STAT3 blockade led to a conspicuous decrease in CXCR4 expression. The expression of STAT3 was dependent on CXCR2 but not CXCR4 (Figure 4(c)).

## 4. Discussion

Timely formation of blood vessels is a prerequisite for the development of bone grafts, including TECs. Increasing evidence suggests that after implantation, most of the donor MSCs die or disappear for a short time [6, 14], putting the indirect paracrine effect of MSCs and the way by which host cells contribute to tissue regeneration into sharper research focus [15]. Among multiple target host cells, EPCs are extensively concerned due to their innate ability of migrating to injured sites to promote angiogenesis and vasculogenesis, a critical step for revascularization. Inspired by this, EPCs have been applied to augment neovascularization in patients with bone injury [16]. Synergistic effects exist between EPCs and MSCs in early revascularization, indicating the importance of EPCs in MSC-induced bone repair [15]. In this study, we echoed the finding that MSCs had a powerful chemotactic effect on EPCs [4, 15]. Moreover, we found that such effect relied on the activation of CXCR2 and CXCR4 in EPCs, both of which were indispensable. This was noteworthy because according to current literature, there had been no report on their equal significance in terms of EPC movement, despite that either CXCR2 or CXCR4 had been identified as a crucial effector.

CXCR2 and CXCR4 are G-protein-coupled receptors (GPCRs). Their interaction with the ligands leads to activation of the associated G protein, which dissociates into the GTP-bound G*α*-subunit and G*βγ*-complex. The *α*-subunit and *βγ*-complex activate multiple pathways to induce different cellular responses, such as adhesion, migration, and chemotaxis [17]. Herein, we found that CXCR4 expression was regulated by CXCR2 and vice versa. These facts indicate that regarding cell migration, CXCR2 and CXCR4 may share certain downstream target signal molecules and there may be a close relationship between them. To date, no evidence is accessible in the field of stem cell therapy. However, findings from tumor research show that CXCL8 stimulation upregulates CXCR4 levels in prostate carcinoma cells and CXCR4 drives tumor invasion and metastasis via activating CXCR2 in breast cancer [18, 19]. In this study, we found that CXCR2 and CXCR4 functioned up- and downstream of each other reciprocally during EPC migration towards MSCs. Intriguingly, the chemotactic effect of both CXCL8 and CXCL12 on EPCs required simultaneous activation of CXCR2 and CXCR4. Activation of CXCR2 or CXCR4 by their respective ligands elicited a positive feedback which in turn increased their own expressions. These findings indicate a new reciprocal crosstalk between CXCR2 and CXCR4 in regulating EPC migration towards MSCs, which can be used for interpreting previous findings that either CXCR2 or CXCR4 is essential for migration of EPCs. Although this phenomenon is described for the first time, it is not uncommon because previous studies suggest that there may be crosstalk or feedback, which contributes to cell migration, between chemokine receptors [20].

To investigate the mode of the crosstalk between CXCR2 and CXCR4, the current literature was reviewed and JAK/STAT3 was selected as a potential connector. The JAK/STAT signaling pathway plays critical roles in bone development and metabolism [21]. For EPCs, STAT3 has been reported to take part in regulating cell survival and proliferation [22, 23]. In response to distinct ligands that bind to GPCRs, JAKs can be activated to stimulate STAT tyrosine phosphorylation [21]. Currently, little is known about the relationship of STAT3 with GPCRs in EPCs, especially in the context of cell migration. Limited hints include that STAT3-deficient cells have a cell-autonomous defect in migration towards CXCR2 ligands [24]. Besides, homodimerization of CXCR4 results in G-protein-independent signaling through the JAK/STAT3 pathway [25]. In this study, we showed that CXCR2 and CXCR4 were regulating targets of STAT3 and cross-activate each other through STAT3 during EPC migration. More evidence supporting the role of STAT3 has been reported previously. While studying CXCR2-mediated neutrophil migration, researchers demonstrate that the gene encoding CXCR2 is a direct STAT3 target. STAT3 regulates CXCR2 expression and functions in cell migration [26]. For CXCR4, phospho-STAT3 has been successfully located in the CXCR4 promoter region, resulting in the activation of CXCR4 transcription and subsequent promotion in MSC motility [27]. Collectively, these further underline the important bridging function of STAT3 between CXCR2 and CXCR4. In addition, our result that the expression levels of CXCR2 and CXCR4 and the chemotaxis of EPCs changed simultaneously after STAT3 inhibition indicated that STAT3 participated in CXCL8- and CXCL12-mediated CXCR2 and CXCR4 activation, as well as EPC migration. Therefore, a feedback loop among CXCR2, CXCR4, and STAT3 induced EPC migration towards MSCs.

*In situ* regenerative medicine approaches have been of special interest since it is widely accepted that the reparative effects of TECs are majorly attributed to host cells [6, 14]. The crosstalk between MSCs and EPCs has been studied previously, but we know little about the mechanism, such as how circulating or tissue-resident EPCs are recruited to implantation sites. However, the relative application has been ongoing for a long time. For example, pharmacological intervention, such as delivery of CXCL12, has been applied to attracting EPCs to ischemic tissue. Moreover, viral transduction strategies have been widely used to introduce specific gene sequences of homing factors prior to implantation, aiming to facilitate *in situ* revascularization [28]. As the techniques that promote precise control over the expression profile of cells are constantly optimized, the reciprocal crosstalk between CXCR2 and CXCR4 in EPCs may be a promising target for developing strategies in the field of *in situ* tissue engineering. In parallel, further in-depth studies employing technologies of genomics and proteomics are expected to unveil the complicated relationship between CXCR2 and CXCR4.

## 5. Conclusions

In summary, the present study demonstrated that MSCs attracted EPCs via simultaneously activating CXCR2 and CXCR4. A new reciprocal crosstalk between CXCR2 and CXCR4 was discovered and this crosstalk linked and acted via STAT3. Activation of the CXCR2-STAT3-CXCR4 loop promoted migration of EPCs. These findings may represent potent targets for developing novel strategies to improve the efficacy of TECs.

## Figures and Tables

**Table 1:** Set up in Transwell chambers.

Upper	hEPCs	hEPC-SB225002
		hEPC-AMD3100
		hEPC-ruxolitinib
		hEPC-CXCR2 shRNA
		hEPC-CXCR4 shRNA
		hEPC-STAT3 shRNA

Lower	Inducing media	BCM
		MSC-CM
		MSC-CM+CXCL8
		MSC-CM+CXCL12
		MSC-CM+CXCL8+CXCL12

hEPCs: human endothelial progenitor cells; BCM: basic culture medium; MSC-CM: conditioned media of mesenchymal stem cells.

**Table 2:** Primers used for RT-PCR.

Gene	Species	Sequence
CXCR2	Human	F: TGCATCAGTGTGGACCGTTA
		R: CCGCCAGTTTGCTGTATTG

CXCR4	Human	F: ATGGAGGGGATCAGTATATACAC
		R: TGGAGTGTGCTATGTTGGCGTCT

GAPDH	Human	F: ATCAACTCACCGCCAACA
		R: CGACTCAATCTTCCTCTCCAG

## Data Availability

The migratory data used to support the findings of this study are included within the article. The protein and gene data used to support the findings of this study are included within the article.

## Supplementary Materials

Supplementary Materials Supplementary Fig. 1 Phenotypic characterization of MSCs by using FACS. Cells were homogenously positive for the cell surface antigens CD44, CD73, CD90 and CD105 and negative for CD14, CD34 and CD45. Supplementary Fig. 2 Phenotypic characterization of EPCs by using FACS. Cells were homogeneously CD34+, CD133+ and VEGFR2+.
