# Supplementary material for: Mesenchymal Stem Cells Attract Endothelial Progenitor Cells via a Positive Feedback Loop between CXCR2 and CXCR4
Source: Stem Cells Int. 2019 Dec 5;2019:4197164. doi: 10.1155/2019/4197164 (PMC6915119; doi:10.1155/2019/4197164)
Supplement: Supplementary Materials — Supplementary Fig. 1 Phenotypic characterization of MSCs by using FACS. Cells were homogenously positive for the cell surface antigens CD44, CD73, CD90 and CD105 and negative for CD14, CD34 and CD45. Supplementary Fig. 2 Phenotypic characterization of EPCs by using FACS. Cells were homogeneously CD34+, CD133+ and VEGFR2+. [file 4197164.f1.pdf]

Supplementary Figure 1

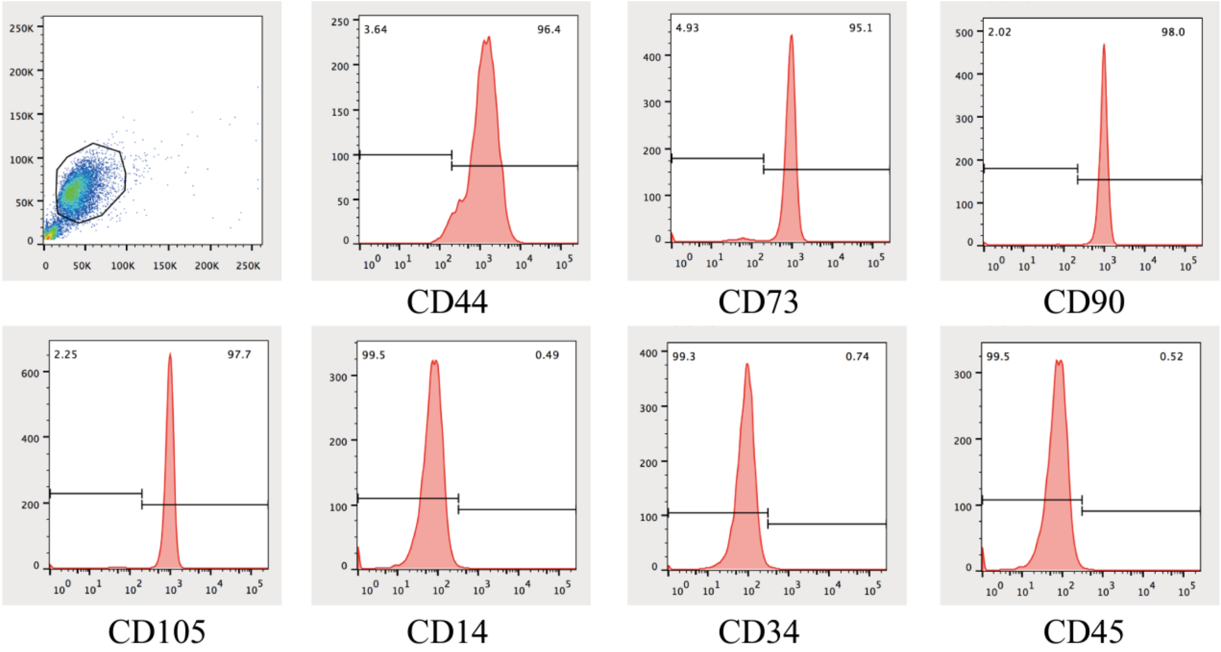

Supplementary Fig. 1 Phenotypic characterization of MSCs by using FACS. Cells were homogenously positive for the cell surface antigens CD44, CD73, CD90 and CD105 and negative for CD14, CD34 and CD45.

Supplementary Figure 2

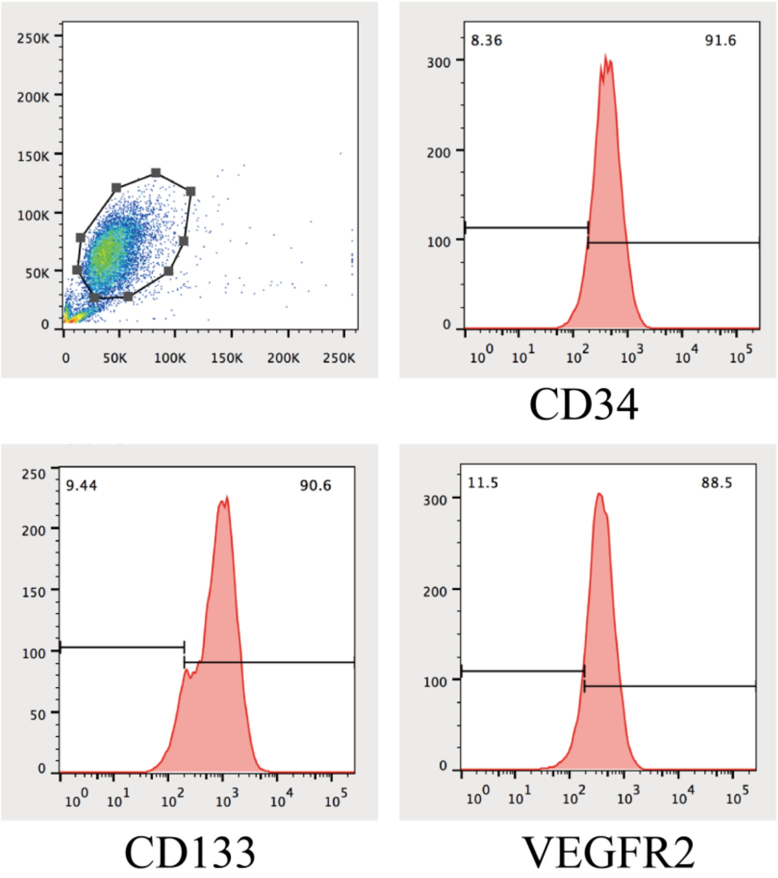

Supplementary Fig. 2 Phenotypic characterization of EPCs by using FACS. Cells were homogeneously $CD34^+$, $CD133^+$ and $VEGFR2^+$.
